# Supplementary material for: Beyond Spin Models in Orbitally Degenerate Open-Shell Nanographenes
Source: Nano Lett. 2024 Oct 7;24(41):12928–34. doi: 10.1021/acs.nanolett.4c03416 (PMC11487622; doi:10.1021/acs.nanolett.4c03416)
Supplement: Supplementary file 1 — nl4c03416_si_001.pdf [file nl4c03416_si_001.pdf]

Supplementary Material of Beyond spin models in orbitally-degenerate open-shell nanographenes

João Henriques
International Iberian Nanotechnology Laboratory (INL), Av. Mestre José Veiga, 4715-330 Braga, Portugal and Universidade de Santiago de Compostela, 15782 Santiago de Compostela, Spain

David Jacob
Departamento de Polímeros y Materiales Avanzados: Física, Química y Tecnología, Universidad del País Vasco UPV/EHU, Av. Tolosa 72, E-20018 San Sebastián, Spain and IKERBASQUE, Basque Foundation for Science, Plaza Euskadi 5, E-48009 Bilbao, Spain

Alejandro Molina-Sánchez
Institute of Materials Science (ICMUV), University of Valencia, Catedrático Beltrán 2, E-46980 Valencia, Spain

Gonçalo Catarina
nanotech@surfaces Laboratory, Empa—Swiss Federal Laboratories for Materials Science and Technology, 8600 Dübendorf, Switzerland

António T. Costa and Joaquín Fernández-Rossier*
International Iberian Nanotechnology Laboratory (INL), Av. Mestre José Veiga, 4715-330 Braga, Portugal
(Dated: August 26, 2024)

I. DENSITY FUNCTIONAL THEORY RESULTS FOR THE AZA-[3]TRIANGULENE DIMER

Density functional theory (DFT) calculations of the Aza-[3]Triangulene (A3T) dimer have been performed using the *ab initio* quantum chemistry package Gaussian16[1], on the level of the generalized gradient approximation (GGA) in the parametrization of Perdew, Burke and Ernzerhof (PBE)[2] together with the 6-311G all-electron triple-ζ basis set. In the initial geometry the carbon and nitrogen atoms are at the carbon sites of an ideal graphene lattice with nearest-neighbor distance of 1.39719Å. The geometry is relaxed for different multiplicities $M = 1, 3, 5, 7$ corresponding to total spins $S_z = (M-1)/2 = 0, 1, 2, 3$. In contrast to Ref. 3 we restrict the relaxation to the flat geometry, i.e., the individual A3T units are not allowed to rotate w.r.t. each other. The reason for only considering a flat geometry here is that we are mainly interested in species adsorbed on flat surfaces. The geometry with lowest energy is encountered for the multiplicity $m = 3$ corresponding to total $S_z = 1$ and $S_{z,1} = S_{z,2} = 1/2$ for the individual spin $S_{z,i}$ of each A3T unit i.

In this geometry we reverse the spin density of one of the A3T units, thus producing an "antiferromagnetic" (AFM) state with $S_{z,2} = -S_{z,1} = -1/2$ and total $S_z = 0$. The relaxed geometry for the AFM state is higher in energy than the relaxed geometry of the "ferromagnetic" (FM) $S_z = 1$ state. Therefore we take the FM geometry as the reference geometry. The DFT calculation of the AFM state in the FM geometry yields an energy 151 meV higher than that of the FM state, as reported in Fig. 2b of the main text together with the spin densities of both FM and AFM solutions. The spin per triangulene unit is $|S_{z,i}| = 0.47$, only slightly lower than the perfect spin-1/2 of the FM solution. A stability analysis of the AFM solution reveals another total $S_z = 0$ state (AFM_2) with an energy lower by 87 meV than the first AFM solution. The AFM_2 solution is thus only 64 meV higher in energy than the FM solution. A comparison of the spin densities of both AFM solutions is shown in Fig. 1. In the AFM_2 solution the spin per A3T unit is actually zero, resulting from finite but opposite spin densities at the borders of each A3T unit.

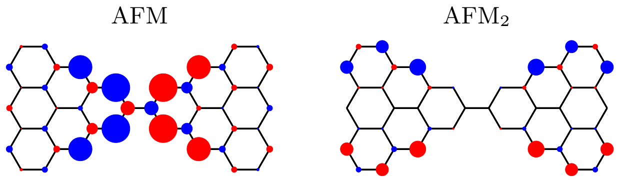

FIG. 1. Spin densities of AFM and AFM_2 solutions of A3T dimer computed in DFT with the PBE functional and the 6-311G basis set. The blue and red circles indicate positive and negative spin density, respectively on each atom. The radius of each circle indicates the magnitude of the spin density.

Finally, we also computed the *charge gap* or *fundamental gap* of the A3T dimer via

$$E_{\rm gap}(N) = E(N+1) + E(N-1) - 2\,E(N) \quad (1)$$

where N is the number of electrons of the neutral species,

$E(N)$ the corresponding GS energy. $E(N \pm 1)$ are the GS energies of the charged species with $N \pm 1$ electrons. We find $E_{\text{gap}}(N) \sim 2.522$eV for the PBE functional and the 6-311G basis set. We also note that the spin of the $N-1$ GS is $S_{N-1} = S_N + \frac{1}{2} = \frac{3}{2}$, while the spin of the $N+1$ GS is $S_{N+1} = S_N - \frac{1}{2} = \frac{1}{2}$.

II. MEAN-FIELD HUBBARD METHOD

Our Mean-Field Hubbard (MFH) calculations consider a tight binding Hamiltonian with first and third neighbor hopping, to which an on-site Hubbard interactions is added

$$H = t \sum_{\langle i,j \rangle,\sigma} c^\dagger_{i,\sigma} c_{j,\sigma} + t_3 \sum_{\langle\langle\langle i,j \rangle\rangle\rangle,\sigma} c^\dagger_{i,\sigma} c_{j,\sigma} + U \sum_i n_{i\uparrow} n_{i\downarrow} \tag{2}$$

where $U > 0$ is the on site Hubbard repulstion and $n_{i\sigma} = c^\dagger_{i\sigma} c_{i\sigma}$ is the number operator for an electron on site i with spin $\sigma = \uparrow, \downarrow$.

Within the collinear MFH approximation, the Hubbard part of the Hamiltonian (a many body operator) is approximated by the single particle Hamiltonian:

$$H^{MF}_U = U \sum_i \langle n_{i\uparrow} \rangle n_{i\downarrow} + n_{i\uparrow} \langle n_{i\downarrow} \rangle + \langle n_{i\uparrow} \rangle \langle n_{i\downarrow} \rangle \tag{3}$$

with $\langle \cdot \rangle$ denoting a thermal average which we compute at zero temperature. We solved the total Hamiltonian in a self-consistent manner, where we start from an initial guess for the occupations $\langle n_{i\sigma} \rangle$, and setting a convergence tolerance of 10^{-8}. To obtain ferromagnetic and antiferromagnetic solutions we use different initial guesses, whose total S_z vanishes if we are looking for the AFM solution, and is finite if we are interested in the FM instead.

III. MEAN-FIELD HUBBARD FOR DIMERS: PHASE DIAGRAM

In this section we shall discuss the stability of the mean field results upon changes on the model parameters for the nitrogen-doped triangulene dimer. In particular, we will probe the parameter space in a section of the plane spanned by V_1 (the on-site potential on the nearest neighbors of the nitrogen atom) and the Hubbard repulsion U. For each pair of these parameters (with fixed hopping parameters and nitrogen on-site potential), we compute the ferromagnetic (FM) and antiferromagnetic (AFM) mean field solutions, and determine their energy difference. The results are summarized in Fig. 2. There we show the energy difference between the FM and AFM solutions for several values of V_1 and U; a negative energy difference implies that the FM solution is more stable than the AFM one. For values of U smaller than and close to $|t|$ we find that the FM solution is always more energetically favorable than the AFM configuration for all the considered values of V_1. However, as U becomes larger than $|t|$ the AFM solution becomes the ground state (the transition is marked in the plot by the dashed line).

Looking at the cross sections depicted in Fig. 2b and c, where we show the energy difference between the FM and AFM solution as a function of U and V_1, respectively, for a fixed value of the other parameter, one sees that depending on the choice of parameters, the FM solution may be the more stable one by a few tens of meV, while the AFM might become the preferred one by a few hundreds of meV.

IV. COMPLETE ACTIVE SPACE (CAS) APPROXIMATION

As opposed to a single-particle problem, the Hubbard Hamiltonian with interactions, i.e. $U \neq 0$, can only be exactly diagonalized for rather small systems, as a consequence of the exponential increase of the Hilbert space as the system size grows. For the molecules we consider in the main text, the Hubbard model with finite U can only be solved using approximate approaches. Here, we make use of the configuration interaction approach combined with the complete active space (CAS) approximation[4]. In this framework, the single-particle problem is solved first. Then, the full Hamiltonian is expressed in terms of the single-particle eigenstates, and N_e electrons are distributed over a subset of N_o orbital close to zero energy (to this we refer as CAS(N_e,N_o)); the remaining electrons fully occupy the orbitals at lower energy (at charge neutrality, $N_e = N_o$). Afterwards, the Hilbert space of all electron configurations of N_e electrons distributed over N_o orbitals is built, and used to diagonalize the CAS Hamiltonian, thus obtaining the approximate energies and wave functions of the Hubbard Hamiltonian.´

V. ADDITIONAL CAS RESULTS

In Fig. 3a we depict the expectation value of S_z at each site of the dimer for the ground state (GS) of the CAS calculation. As mentioned in the main text, this magnetization map is in good agreement with the analogous results found from DFT and MFH calculations.

In Fig. 3b we show the charge maps at each site of the dimer, $n_{i,\uparrow} + n_{i,\downarrow}$, for the first 4 triplets and first 4 singlets of the CAS calculation. The fact that the charge map for the least energetic states is different for the triplet and the singlet, reflects the spin-orbital interplay: different charge maps imply different occupation of the molecular orbitals. Further evidence for the spin-orbital interplay comes from the fact that the charge map of the $S = 0$ lowest energy state is the same than the charge map of the $S = 1$ highest energy state.

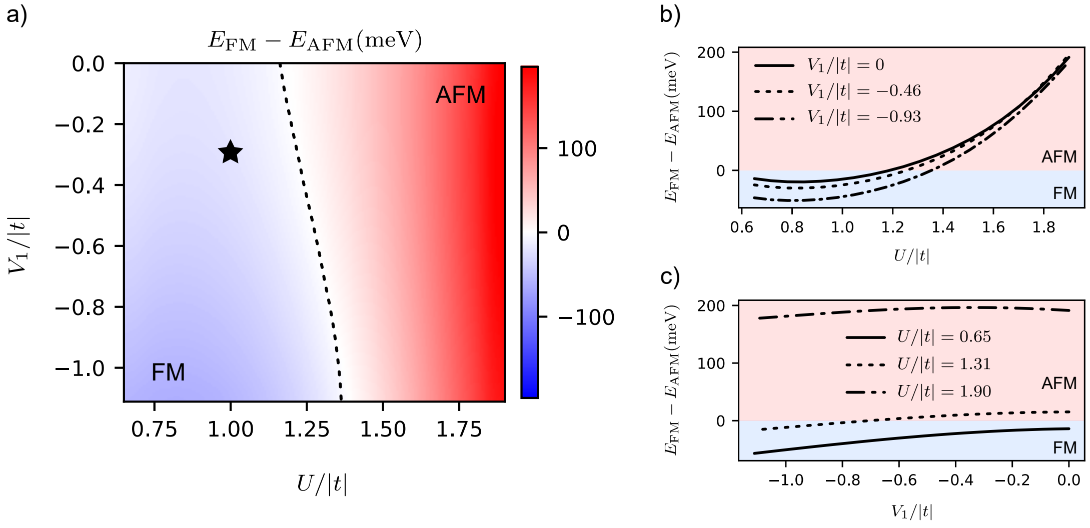

FIG. 2. a) Color map of the energy difference between the FM and the AFM solution of the mean field calculation as a function of the Hubbard repulsion U and the on-site potential of on the first neighboring sites of the nitrogen atom V_1; both parameters are shown as a function of the first neighbor hopping $|t| = 2.7$ eV. The dashed line marks the points where $E_{\rm FM} - E_{\rm AFM} = 0$; the star indicates the region of the parameter space considered in the main text, chosen to obtain bands of 2D crystal in agreement with DFT. Panels b) and c) show cross sections of the color map of panel a).

VI. DENSITY FUNCTIONAL THEORY FOR CRYSTALS

The DFT calculations have been performed at the level of the generalized gradient approximation (GGA) using the PBE functionals. The pseudopotentials are norm-conserving with core correction, and generated with the ONCVPSP code[5]. The plane waves basis set is defined by a energy cutoff of 70 Ry. The total energies are converged with a $\mathbf{k}$−point grid of $15 \times 15 \times 1$ and a smearing of 1 meV.

VII. NON-MAGNETIC AND ANTIFERROMAGNETIC PHASES FOR THE CRYSTALS

In Fig. 4 we present a comparison between the energy bands obtained with DFT and the Hubbard model in the self-consisted mean-field approximation. We focus here on the antiferromagnetic and non-magnetic configurations. The ferromagnetic configuration (which has the lowest energy of the three) is discussed in the main text. In the mean-field calculation, different self-consistent configurations with local moments and a vanishing global magnetization can be reached by choosing appropriate initial guesses for the magnetization distribution. For instance, if the initial magnetization is set to zero in every atomic site, the self-consistent mean-field solution will converge to a non-magnetic state. We also note that in the case of the A3T, the charge distributions obtained with mean-field calculations are inhomogeneous, as shown in Fig. 5, due to the different on-site potentials at carbon and nitrogen sites.

U (eV)	$E_{\rm FM} - E_{\rm AF}$ (meV)	S_z/triang, AF	FM splitting (meV)
2.7	-67	0.20	290
3.0	-68	0.33	330
3.3	-100	0.40	380
4.0	-87	0.49	508

TABLE I. Mean-field results for the 2D crystal as a function of the Hubbard U, for fixed tight-binding parameters. The last column refers to the energy difference between equivalent point in the spin-up and spin-down FM bands.

In choosing the value for the strength of the on-site repulsion U in the calculations based on the Hubbard model, we did not try to perform any kind of fine tuning. The adoption of $U = |t|$ is consistent with the recent literature on magnetism in carbon systems, including previous publications by the authors. It is worth noting, however, that changes in U can produce changes in quantities like the energy difference between FM and AFM configurations, or magnetic moment per triangulene, without changing the overall qualitative picture. In Table I we present results for some values of U close to the one we chose. Notice that the FM-AFM energy difference and the spin per triangulene in the AFM configuration are both in good agreement with the DFT results (shown in the main text) for $U = 3.3$ eV. The qualitative behavior of the system, however, is essentially unchanged by this choice.

In Fig. 6 we compare the spatial distribution of the magnetization for the ferromagnetic and the antiferromagnetic configurations of the 2D A3T crystal. As stated in the main text, the AFM configuration can not be ob-

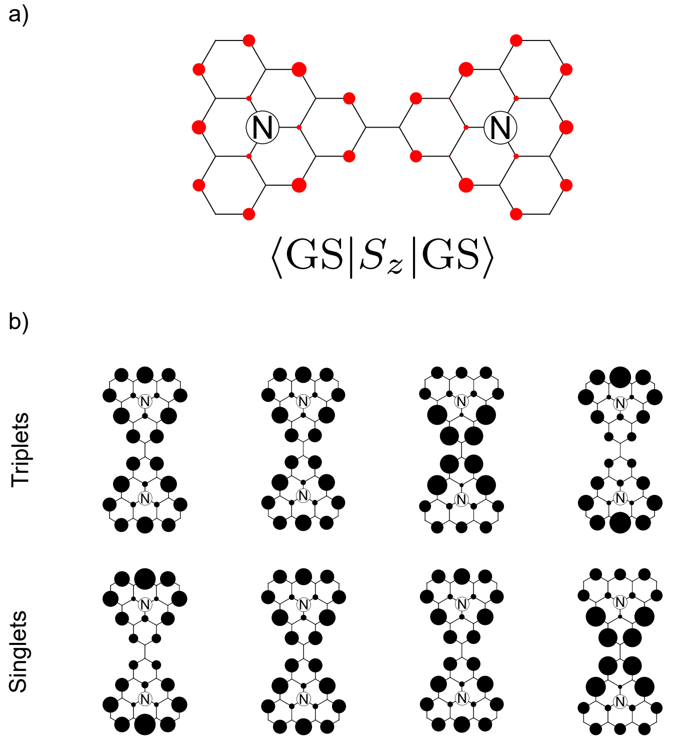

FIG. 3. a) Expectation value of the local S_z operator at each site, computed over the $S_z = +1$ ground state (GS) of the CAS calculation; b) Charge maps for the first four triplets and first four singlets of the CAS calculations (arranged in increasing energy order from left to right).

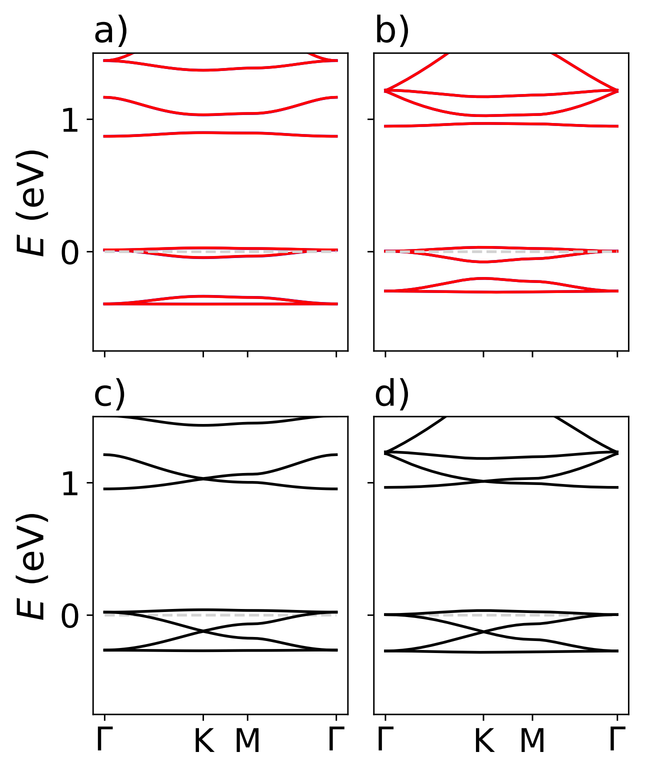

FIG. 4. Energy bands for A3T for the antiferromagnetic (panels **a** and **b**) and non-magnetic (panels **c** and **d**) configurations obtained with DFT (panels **a** and **c**) and mean-field Hubbard model (panels **b** and **d**). Spin-up and spin-down bands are degenerate. For the mean-field Hubbard model calculation we adopted $U = |t|$.

tained from the FM configuration by simply flipping the spins of one of the triangulenes, although this difference is much smaller than in the case of the dimer.

VIII. CALCULATION OF THE MAGNON SPECTRAL DENSITY FROM THE HUBBARD MODEL

In the fermionic language of the Hubbard model, magnons are associated with spin-flip excitations encoded by the operator

$$S^-(\vec{R}) \equiv c^\dagger_\downarrow(\vec{R}) c_\uparrow(\vec{R}), \qquad (4)$$

where $c^\dagger_\sigma(\vec{R})[c_\sigma(\vec{R})]$ creates [annihilates] an electron at lattice position $\vec{R}$ with spin σ. The so-called transverse spin susceptibility

$$\chi^{+-}(\vec{R}, \vec{R}'; t) \equiv -i\theta(t) \left\langle \left[S^+(\vec{R}, t), S^-(\vec{R}', 0) \right] \right\rangle, \qquad (5)$$

plays the role of the magnon Green function, by analogy to the one-electron Green function.[6] This two-time correlation function cannot be computed exactly for an interacting model such as the one represented by the Hubbard Hamiltonian. The simplest approach that can describe magnons is the so-called random phase approximation (RPA), in which the interaction is taken into account, to all orders in perturbation theory, between the electron and the hole that form the spin-flip excitation.[6] The RPA relates the interacting susceptibility to the mean-field susceptibility calculated for the lowest energy broken-symmetry configuration of the model in question,[7]

$$\chi^{+-}_{\rm MF}(\vec{R}, \vec{R}; t) \equiv -i\theta(t) \left\langle \left[S^+(\vec{R}, t), S^-(\vec{R}', 0) \right] \right\rangle_{\rm MF}, \qquad (6)$$

via the equation

$$\chi^{+-}_{\rm RPA}(\vec{R}, \vec{R}'; \hbar\Omega) = \chi^{+-}_{\rm MF}(\vec{R}, \vec{R}'; \hbar\Omega) + \sum_{\vec{R}''} \chi^{+-}_{\rm MF}(\vec{R}, \vec{R}'', \hbar\Omega) U(\vec{R}'') \chi^{+-}_{\rm RPA}(\vec{R}'', \vec{R}'; \hbar\Omega), \qquad (7)$$

where

$$\chi^{+-}(\vec{R}, \vec{R}'; \hbar\Omega) \equiv \int_{-\infty}^{\infty} dt\, e^{-i\Omega t} \chi^{+-}(\vec{R}, \vec{R}', t) \qquad (8)$$

and $U(\vec{R})$ is the strength of the intra-site Coulomb repulsion encoded in the Hubbard term. For systems with translation symmetry but multi-atom unit cells, like the triangulene crystals investigated in this letter, the transverse spin susceptibility can be expressed as a matrix in

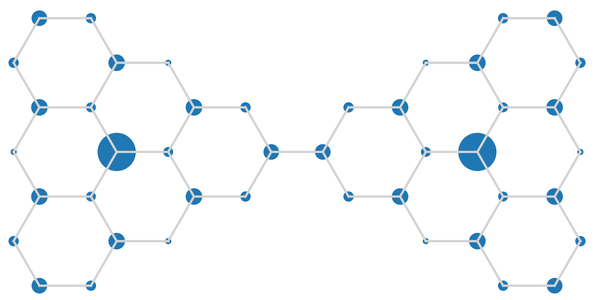

FIG. 5. Spatial distribution of charge in the unit cell of the A3T crystal in the non-magnetic configuration. The radii of the circles represent the deviation from the average occupancy of 23/22 electrons per site.

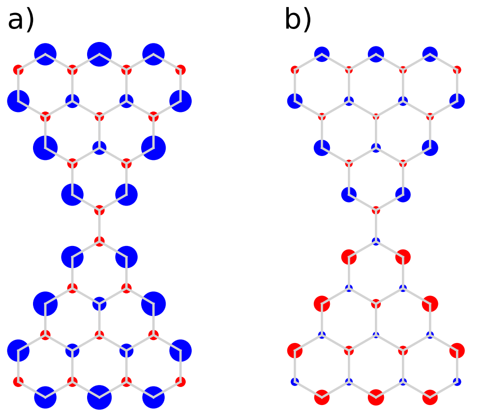

FIG. 6. Spatial distribution of the magnetization within a unit cell of the 2D A3T crystal, for the ferromagnetic (**a**) and antiferromagnetic (**b**) configurations. The radii of the discs represent the absolute value of the magnetization at each site, and the colors represent the two possible magnetization directions. The total magnetization per triangulene in the FM configuration is $0.98\mu_B$, whereas in the AFM configuration it is $0.41\mu_B$

intra-cell atomic site indices, whose elements are functions of a wave vector $\vec{Q}$ that spans the Brillouin zone of the crystal,

$$\chi^{+-}_{ll'}(\vec{Q};\hbar\Omega) \equiv \frac{1}{N}\sum_{\vec{R}} e^{-i\vec{Q}\cdot\vec{R}}\chi^{+-}(\vec{R}+\vec{r}_l,\vec{r}_{l'};\hbar\Omega), \quad (9)$$

where $\vec{r}_l$ is the position of atom l within the unit cell with respect to the unit cell origin $\vec{R}$. The magnon spectral density at wave vector $\vec{Q}$ and energy $\hbar\Omega$, projected on site l of the unit cell is given by

$$A_l(\vec{Q};\hbar\Omega) \equiv -\frac{1}{\pi}\text{Im}\chi^{+-}_{ll}(\vec{Q};\hbar\Omega). \quad (10)$$

Typically, for a fixed wave vector $\vec{Q}$, the magnon spectral density as a function of energy has several peaks, associated with magnon eigenmodes. By systematically mapping the peak energies for each wave vector along a given path within the crystal Brillouin zone, it is possible to construct magnon dispersion relations, such as shown in Fig. 4 of the main text. Another important piece of information contained in the spectral density is the width of the magnon peaks. For insulating magnets the magnon peaks have vanishing linewidth, indicating that the magnon modes are stationary states of the Hubbard Hamiltonian. This also means that the low energy dynamics of the system are well described by a spin-only Hamiltonian, such as the Heisenberg model. In contrast, for metallic magnets the magnon peaks have finite linewidths, which increase monotonically with increasing magnon energy. This is a result of the coupling between magnons and the continuum of incoherent spin flip modes known as Stoner excitations. This coupling, besides endowing magnons with finite lifetimes, renormalizes their energies. Neither the finite lifetime nor the energy renormalization can be captured by a spin-only Hamiltonian. This is exactly what we observe for the magnons of the nitrogen doped triangulenes reported in this letter, as shown in Fig. 4 of the main text.

Finally, we would like to address the issue of the number of magnon modes that can be identified at low energies for triangulene crystals. In general, the number of magnon modes expected from a system with N magnetic sites within the unit cell is exactly N. It is common, however, to find systems in which the lowest energy modes are well separated from the higher energy ones. In this case it is safe to interpret the lowest modes as "rigid" motions of a few "macro-"spins (the precise number of modes and "macro-"spins depend on the geometry of the unit cell) which can then be used to build effective spin-only models. This is the case for carbon-only triangulenes, as some of the authors have shown in ref. 8. In that case, the magnon spectra clearly show two modes at low energies (degenerate in the case of the antiferromagnetic crystals). Combined with the geometry of the unit cell (formed by two triangulenes), this naturally suggests that the low energy dynamics of those systems are well described by assigning an effective "macro-"spin to each triangulene. This spin-only description is further validated by the fact that the magnon peaks in the spectral densities have vanishing linewidths. In contrast, the magnon spectrum of the nitrogen doped triangulenes reported here display at least three well defined peaks within a relatively narrow energy window, with aditional (broader) peaks seen at slightly higher energies. This indicates that the magnon modes of the N-doped triangulenes involve spatially inhomogeneous fluctuations of the spin densities within each triangulene, thus prohibiting a

"macro-"spin description of their low-energy dynamics.

---

* On permanent leave from Departamento de Física Aplicada, Universidad de Alicante, 03690 San Vicente del Raspeig, Spain.

[1] M. J. Frisch *et al.*, Gaussian 16 Revision C.01 (2016), Gaussian Inc. Wallingford CT.

[2] John P. Perdew, Kieron Burke, and Matthias Ernzerhof, "Generalized gradient approximation made simple," Phys. Rev. Lett. **77**, 3865–3868 (1996).

[3] Hongde Yu and Thomas Heine, "Magnetic coupling control in triangulene dimers," J.Am. Chem. Soc. **145**, 19303 (2023).

[4] Ricardo Ortiz, Roberto Álvarez Boto, Noel García-Martínez, Juan Carlos Sancho-García, Manuel Melle-Franco, and Joaquín Fernández-Rossier, "Exchange rules for diradical π-conjugated hydrocarbons," Nano Lett. **19**, 5991–5997 (2019).

[5] D. R. Hamann, "Optimized norm-conserving vanderbilt pseudopotentials," Phys. Rev. B **88**, 085117 (2013).

[6] S. Doniach and E.H. Sondheimer, *Green's Functions for Solid State Physicists* (Imperial College Press, 1998).

[7] L. H. M. Barbosa, R. B. Muniz, A. T. Costa, and J. Mathon, "Spin waves in ultrathin ferromagnetic overlayers," Phys. Rev. B **63**, 174401 (2001).

[8] G. Catarina, J. C. G. Henriques, A. Molina-Sánchez, A. T. Costa, and J. Fernández-Rossier, "Broken-symmetry magnetic phases in two-dimensional triangulene crystals," Phys. Rev. Res. **5**, 043226 (2023).
